# CIB1 protects against MPTP-induced neurotoxicity through inhibiting ASK1

**DOI:** 10.1038/s41598-017-12379-3

**Published:** 2017-09-22

**Authors:** Kyoung Wan Yoon, Hyun-Suk Yang, Young Mok Kim, Yeonsil Kim, Seongman Kang, Woong Sun, Ulhas P. Naik, Leslie V. Parise, Eui-Ju Choi

**Affiliations:** 1 Department of Life Sciences, Korea University, Seoul, 02841 Korea; 2 Department of Biotechnology, Hoseo University, Asan, 31499 Korea; 3 Department of Anatomy, Brain Korea 21 Program, Korea University College of Medicine, Seoul, 02841 Korea; 4 Cardeza Center for Vascular Biology, Department of Medicine, Thomas Jefferson University, Philadelphia, PA 19107 USA; 5 Department of Biochemistry and Biophysics, University of North Carolina at Chapel Hill, Chapel Hill, NC 27599 USA

## Abstract

Calcium and integrin binding protein 1 (CIB1) is a calcium-binding protein that was initially identified as a binding partner of platelet integrin α_IIb_. Although CIB1 has been shown to interact with multiple proteins, its biological function in the brain remains unclear. Here, we show that CIB1 negatively regulates degeneration of dopaminergic neurons in a mouse model of Parkinson’s disease using 1-methyl-4-phenyl-1,2,3,6-tetrahydropyridine (MPTP). Genetic deficiency of the CIB1 gene enhances MPTP-induced neurotoxicity in dopaminergic neurons in CIB1^−/−^ mice. Furthermore, RNAi-mediated depletion of CIB1 in primary dopaminergic neurons potentiated 1-methyl-4-phenyl pyrinidium (MPP^+^)-induced neuronal death. CIB1 physically associated with apoptosis signal-regulating kinase 1 (ASK1) and thereby inhibited the MPP^+^-induced stimulation of the ASK1-mediated signaling cascade. These findings suggest that CIB1 plays a protective role in MPTP/MPP^+^-induced neurotoxicity by blocking ASK1-mediated signaling.

## Introduction

Parkinson’s disease is a neurodegenerative disease characterized by the selective loss of dopaminergic neurons in the substantia nigra pars compacta (SNpc) and their targets in the striatum^1–3^. Although the molecular mechanism underlying the loss of SNpc neurons is not clearly understood, mitochondrial dysfunction with a defect of complex I appears to be one of the key features in the pathogenesis of Parkinson’s disease^2,4^. Dysfunction of complex I results in enhanced production of reactive oxygen species (ROS), thereby causing oxidative stress^5^. Massive ROS generation induces cell death signaling pathways, including stress-activated protein kinase pathways such as the c-Jun NH_2_-terminal kinase (JNK) pathway. JNK activation in dopaminergic neurons has been shown in animal models of Parkinson’s disease as well as in human patients^6,7^. Furthermore, a JNK inhibitor exhibits a neuroprotective effect in animal models of the disease^8,9^. Apoptosis signal-regulating kinase 1 (ASK1), a mitogen-activated protein kinase kinase kinase (MAP3K) in the JNK pathway, is also activated in a mouse model of Parkinson’s disease and mediates cell death of dopaminergic neurons^10,11^.

CIB1 is a calcium-binding protein of 22 kDa, containing two canonical EF-hand domains in the carboxyl-terminal globular region^12,13^. CIB1, which was originally identified as a calcium and integrin binding protein^14^, appears to be involved in the regulation of various physiological and pathological processes including spermatogenesis^15^, ischemia-induced angiogenesis^16^, and cardiac hypertrophy^17^. CIB1 interacts with a number of proteins, including Rac3 and several serine/threonine kinases such as polo-like kinases, focal adhesion kinase, and p21-activated kinase^18–21^. It also physically associates with and inhibits ASK1, thereby preventing stress-induced apoptosis in a calcium ion-sensitive manner^22^. CIB1 is ubiquitously expressed in various cell types including neurons in the brain^14,19,23,24^. In particular, CIB1 in the brain has been reported to be involved in microtubule dynamics during neurite outgrowth^25^, synaptic plasticity through interacting with the polo-like kinases Fnk and Snk in hippocampal neurons^19^, and neuronal development with targeting NBR1 and FEZ1 in neural tube^26^. However, its biological function in the neuronal system remains unclear.

We previously showed that CIB1 negatively regulates the stress-induced apoptosis of cultured dopaminergic neurons^22^. In order to better understand a regulatory function of CIB1 in the pathogenesis of Parkinson’s disease, we have now investigated the inhibitory action of CIB1 against dopaminergic neurotoxicity in a mouse model of the disease using 1-methyl-4-phenyl-1,2,3,6-tetrahydropyridine (MPTP). MPTP is a neurotoxin that causes Parkinson’s disease in humans and vertebrates that is clinically almost indistinguishable from sporadic Parkinson’s disease^27^. Here, we show that ablation of the CIB1 gene in mice increased the susceptibility of dopaminergic neurons to MPTP toxicity. Moreover, CIB1 inhibited the stimulation of ASK1 activity induced by 1-Methyl-4-phenylpyridinium (MPP^+^), the toxic metabolite of MPTP. Collectively, our findings suggest that CIB1 mitigates MPTP/MPP^+^-induced neurotoxicity by targeting ASK1.

## Results

### Genetic ablation of CIB1 in mice potentiates MPTP-induced dopaminergic neurotoxicity

**Figure 1:**
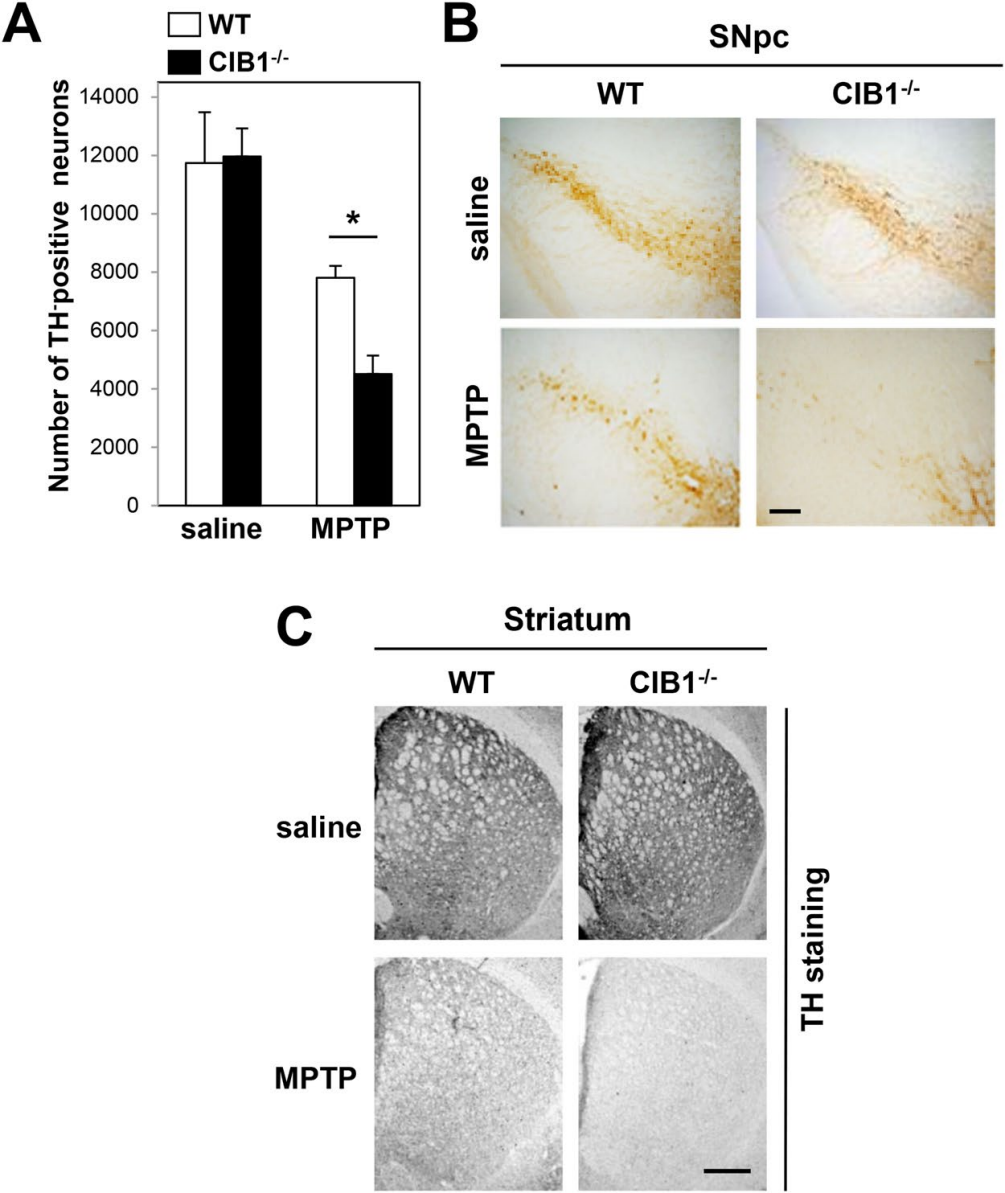
CIB1 deficiency potentiates MPTP-induced loss of dopaminergic neurons. (**A**) Wild-type (WT) and CIB1^−/−^ mice (2~3-month-old group) were treated with MPTP (30 mg/kg every 24 h, five times) or saline by intraperitoneal injection. Immunohistochemical staining for TH was performed on coronal brain sections from WT and CIB1^−/−^ mice. Numbers of TH-positive neurons in the substantia nigra pars compacta were analyzed by stereological counting. Data are means ± SD (n = 5). *P < 0.05. (**B**,**C**) The representative images of TH-stained neurons in substantia nigra pars compacta (**B**) or striatum (**C**) of WT and CIB1^−/−^mice were shown. Scale bars, 100 μm (**B**) and 250 μm (**C**).

CIB1 was previously shown to reduce dopaminergic neuronal death induced by 6-hydroxydopamine, a widely used neurotoxin in experimental models of Parkinson’s disease^22^. To better understand the *in vivo* function of CIB1 in the pathogenesis of Parkinson’s disease, we examined the effect of CIB1 gene deletion on loss of dopaminergic neurons in the SNpc in a MPTP mouse model of the disease. Immunohistochemical staining for tyrosine hydroxylase (TH) and stereological analysis revealed that MPTP treatment resulted in a more severe loss of TH-positive dopaminergic neurons in the SNpc of CIB1-knockout (CIB1^−/−^) mice, compared to that of wild-type mice (Fig. 1A,B). Furthermore, MPTP administration reduced the TH-positive fibers in the striatum in wild-type mice, and this reduction was further aggravated by ablation of the CIB1 gene in CIB1^−/−^ mice (Fig. 1C).

### CIB1 depletion by RNA interference potentiates MPP^+^-induced dopaminergic neuronal death

**Figure 2:**
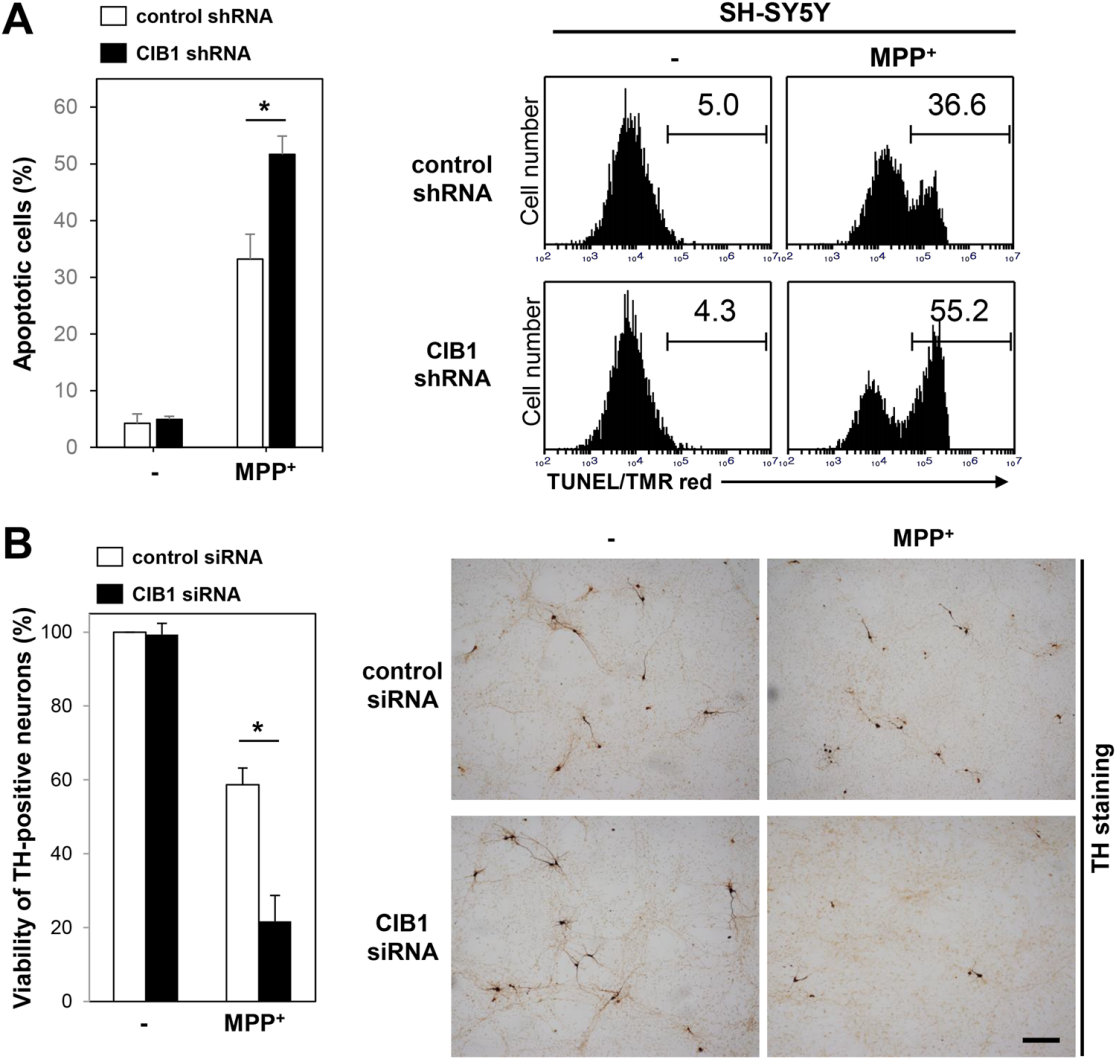
CIB1 mitigates MPP^+^-induced neurotoxicity in dopaminergic neurons. (**A**) SH-SY5Y cells expressing either control (GFP) or CIB1 shRNA were incubated for 20 h in the absence or presence of 3 mM MPP^+^. Then, the cells were stained with red fluorescein (TMR red)-labeled TUNEL and analyzed by flow cytometry. The apoptosis was determined by TUNEL-positive population showing positively shifted TMR red signal. The percents of TUNEL-positive cells are indicated as apoptotic population. Representative data of apoptosis are shown in the right panel. The left graph represents mean ± SD calculated from triplicate experiments (n = 3). *P < 0.05. (**B**) Primary mesencephalic neurons in culture were transfected for 48 h with control (GFP) or CIB1 siRNA. The neurons were left untreated or treated with 200 μM MPP^+^ for 20 h, and then were subjected to immunocytochemistry with anti-TH antibody. The representative images of the stained cells are shown in the right panels (scale bar, 100 μm), and the viability of TH-positive neurons is shown in the left panel as a percentage of that for control siRNA-transfected untreated cells. Data are means ± SD of values from three independent experiments. *P < 0.05.

Monoamine oxidase B (MAO-B) in the brain converts MPTP to a toxic metabolite, MPP^+^. MPP^+^ causes mitochondrial dysfunction by inhibiting complex I of the electron transport chain, leading to oxidative stress-induced cell death^28,29^. In our initial experiments, we examined the effect of CIB1 on MPP^+^-induced apoptotic cell death in human neuroblastoma SH-SY5Y cells. MPP^+^ increased apoptotic cell death in these cells, and this effect was further enhanced by RNA interference (RNAi)-mediated depletion of CIB1 (Fig. 2A, Fig. S1A). Furthermore, forced expression of CIB1 in SH-SY5Y cells reduced the MPP^+^-induced apoptosis (Fig. S2). Next, we examined the effect of CIB1 on MPP^+^-induced neurotoxicity in a primary culture of rat mesencephalic dopaminergic neurons. Immunocytochemical analysis of TH-positive neurons revealed that MPP^+^ reduced viability of mesencephalic dopaminergic neurons, and this toxic effect was further potentiated by siRNA-mediated depletion of CIB1 (Fig. 2B, Fig. S1B). Together, these results suggest that CIB1 mitigates MPP^+^-induced neurotoxicity in dopaminergic neurons.

### CIB1 negatively regulates the ASK1-JNK signaling pathway

MPP^+^ treatment enhances ROS production, which is critical for MPP^+^-induced apoptosis^30,31^. Therefore, we examined whether CIB1 could modulate MPP^+^-induced ROS generation. As expected, MPP^+^ treatment resulted in an increase in ROS production in SH-SY5Y cells, and this increase was not changed by RNAi-mediated depletion of CIB1 expression in these cells (Fig. S3). These results suggest that CIB1 does not affect MPP^+^-induced ROS production.

**Figure 3:**
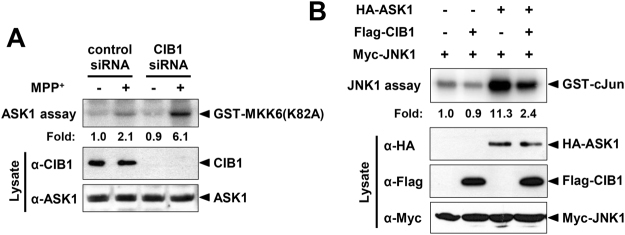
CIB1 inhibits the MPP^+^-induced stimulation of ASK1 in primary dopaminergic neurons. (**A**) Primary mesencephalic neurons were transfected for 48 h with either control (GFP) or CIB1 siRNA. The neurons were incubated in the absence or presence of 200 μM MPP^+^ for 2 h. Cell lysates were subjected to immunoprecipitation with anti-ASK1 antibody. The resulting precipitates were assayed for ASK1 activity using substrates GST-MKK6 (K82A). The lysates were also examined directly by immunoblot analysis with antibodies to CIB1 or ASK1. (**B**) SH-SY5Y cells were transfected for 48 h with the indicated combinations of expression vectors for HA-ASK1, Flag-CIB1, and Myc-JNK1. The cells were then lysed and subjected to immunoprecipitation with anti-Myc antibody, and the resulting precipitates were assayed for JNK1 activity.

CIB1 has been shown to inhibit ASK1-dependent signaling events^22^. Given that ASK1 mediates MPP^+^-induced neuronal death and MPTP-induced Parkinsonism^6,10,32,33^, we investigated whether CIB1 could regulate MPP^+^-induced ASK1 signaling. MPP^+^ treatment induced the stimulation of ASK1 in rat primary mesencephalic dopaminergic neurons, and this stimulation was potentiated by siRNA-mediated depletion of CIB1 expression in these cells (Fig. 3A). Thus, these results indicated that CIB1 negatively regulates the MPP^+^-induced stimulation of ASK1. Furthermore, forced expression of CIB1 inhibited the ASK1-induced activation of JNK in SH-SY5Y cells transfected with vectors for Flag-CIB1, HA-ASK1, and Myc-JNK1 (Fig. 3B), suggesting that CIB1 inhibits the ASK1-induced stimulation of JNK1.

### CIB1 physically interacts with ASK1

**Figure 4:**
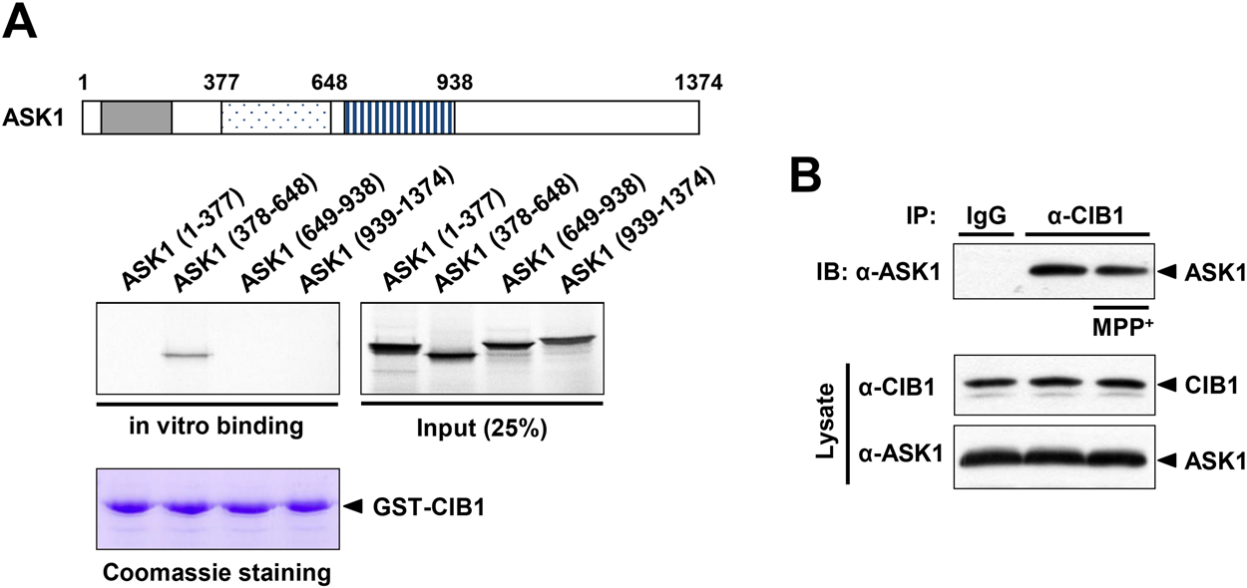
CIB1 physically associates with ASK1 in mesencephalic dopaminergic neurons. (**A**) A schematic representation of ASK1 indicating the thioredoxin-binding region (gray box), the TRAF2-binding region (dotted box), and the kinase domain (hatched box) is shown in the upper panel. The indicated ^35^S-labled ASK1 variants were produced by *in vitro* translation and incubated for 4 h at 4 °C with GST-CIB1 immobilized on glutathione-agarose beads. The bead-bound ^35^S-labled proteins were eluted and detected by SDS-PAGE and autoradiography. The gel was also stained with Coomassie Brilliant Blue. A portion (25%) of the ^35^S-labled protein input to the binding reaction was also directly subjected to SDS-PAGE and autoradiography. (**B**) Primary mesencephalic neurons in culture were incubated in the absence or presence of 200 μM MPP^+^ for 2 h, lysed, and subjected to immunoprecipitation (IP) with antibodies to ASK1 (α-ASK1) or with rabbit preimmune IgG (control). The resulting precipitates as well as cell lysates were subjected to immunoblot (IB) analysis with antibodies to CIB1 or to ASK1.

CIB1 interacts with ASK1^22^. Indeed, *in vitro* binding assay data revealed that glutathione S-transferase (GST)-fused CIB1 physically associated with a deletion mutant of ASK1 consisting of amino acids 378 to 648 [ASK1 (378-648)] (Fig. 4A). ASK1 (378–648) contains the amino-terminal regulatory domain^34^, which is also responsible for the binding of ASK1 to TRAF2^35^. Next, we examined whether MPP^+^ could modulate the interaction between CIB1 and ASK1. Co-immunoprecipitation analysis data indicated that CIB1 interacted with ASK1 in primary mesencephalic neurons (Fig. 4B), and that this interaction was not affected by MPP^+^ treatment.

### CIB1 blocks the binding of TRAF2 to ASK1 and the phosphorylation of ASK1 on Thr^845^

**Figure 5:**
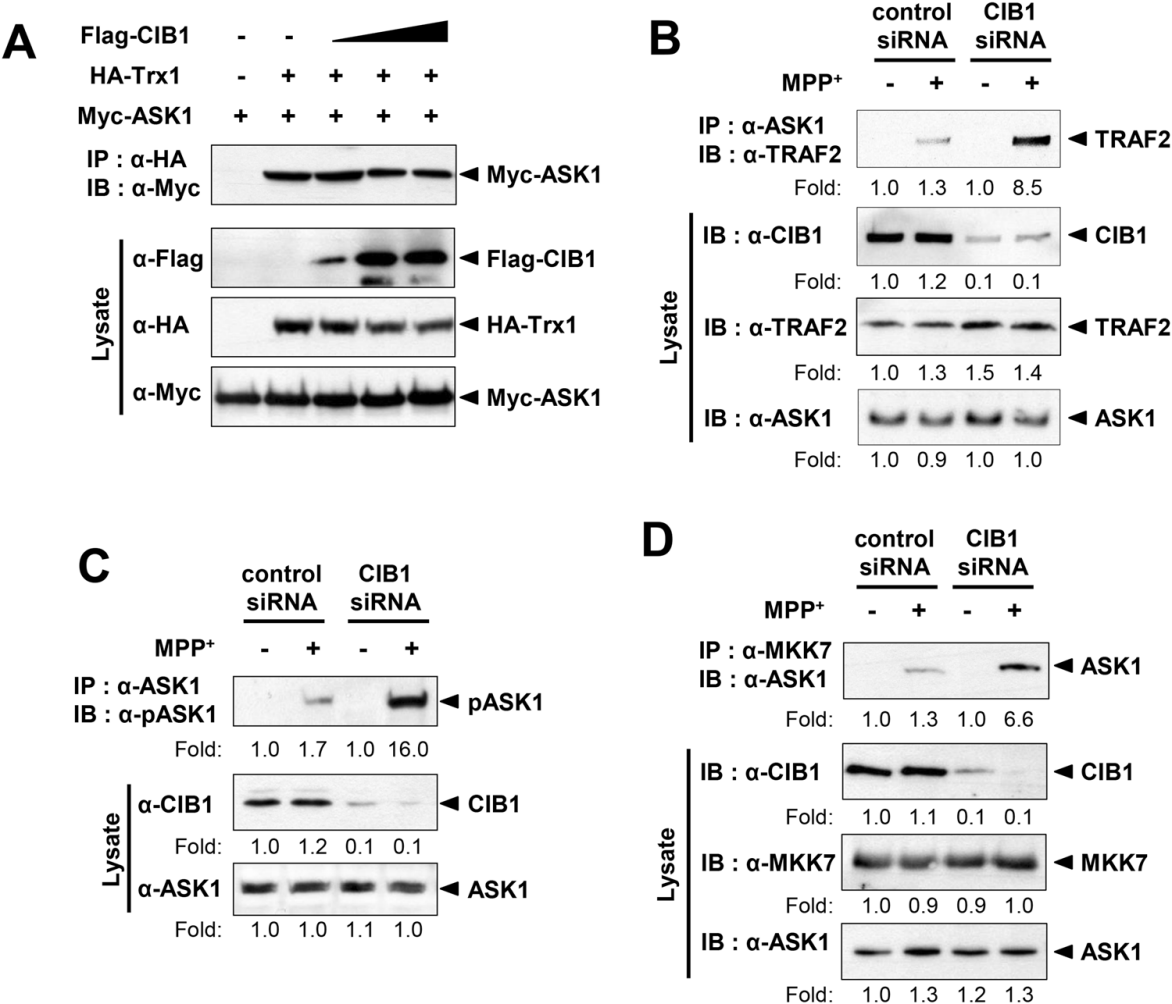
CIB1 inhibits the binding of TRAF2 to ASK1 and the phosphorylation of ASK1 on Thr^845^ in intact cells. (**A**) SH-SY5Y cells were transfected for 48 h with the indicated combinations of vectors encoding Flag-CIB1, HA-Trx1, and Myc-ASK1. Cell lysates were subjected to immunoprecipitation with anti-HA antibody. The resulting precipitates were subjected to immunoblot analysis with anti-Myc antibody. Cell lysates were also examined directly by immunoblot analysis with the indicated antibodies. (**B**,**C**,**D**) Primary mesencephalic neurons in culture were transfected with either control or CIB1 siRNA for 48 h. The neurons were left untreated or treated with 200 μM MPP^+^ for 2 h, lysed, and subjected to immunoprecipitation with antibody to ASK1 (**B**,**C**) or antibody to MKK7 (**D**). The resulting precipitates were immunoblotted with antibodies to ASK1. Cell lysates were also immunoblotted directly with antibodies to CIB1, to ASK1, or to MKK7. The levels of protein expression are determined by relative fold of band image intensity (the first lane, fold 1.0).

ASK1 in a basal state appears to form a complex with the reduced form of the redox protein thioredoxin-1 (Trx1), which keeps ASK1 inactive^36^. When oxidative stress enhances cellular ROS generation, Trx1 is oxidized and dissociates from ASK1. Subsequently, TRAF2 is recruited to free ASK1 and promotes the autophosphorylation of ASK1 at a threonine residue in the catalytic domain (Thr^838^ or Thr^845^ in the human or mouse ASK1, respectively), resulting in ASK1 activation^36–38^. In order to understand the mechanism by which CIB1 inhibits ASK1 activation, we examined whether CIB1 could affect the interaction between ASK1 and Trx1. Co-immunoprecipitation analysis revealed that CIB1 did not change the interaction between Trx1 and ASK1 (Fig. 5A). Next, we tested whether CIB1 could affect the MPP^+^-induced binding of ASK1 to TRAF2 in primary mesencephalic neurons. MPP^+^ induced the interaction between ASK1 and TRAF2 in the neurons, which was further enhanced by CIB1 depletion (Fig. 5B). These results suggest that CIB1 negatively regulates MPP^+^-induced recruitment of TRAF2 to ASK1. It is noteworthy that the CIB1-binding region of ASK1 as shown in Fig. 4A overlaps with the TRAF2-binding region of the kinase^35^. Next, we examined the effect of CIB1 on the MPP^+^-induced phosphorylation of ASK1 at Thr^845^. MPP^+^ treatment increased ASK1(Thr^845^) phosphorylation in primary mesencephalic neurons, and this increase was further enhanced by CIB1 depletion (Fig. 5C). We also examined the action of CIB1 on the MPP^+^-induced interaction between ASK1 and MKK7. MKK7 is a mitogen-activated protein kinase kinase (MAP2K), acting as the downstream kinase of ASK1, in the JNK pathway^39,40^. Our results revealed that depletion of CIB1 enhanced the interaction between ASK1 and MKK7 in CIB1 siRNA-transfected cells (Fig. 5D), suggesting that CIB1 negatively regulates the binding of ASK1 to its substrate MKK7. Consistent with these results, ectopically expressed CIB1 inhibited the interaction between ASK1 and MKK7 in transfected SH-SY5Y cells (Fig. S4). Together, our results suggest that CIB1 inhibits the stimulating effect of MPP^+^ on the recruitment of TRAF2 to ASK1, ASK1(Thr^845^) phosphorylation, and the binding of ASK1 to its substrate, MKK7, in primary mesencephalic neurons. Of note, the abundance of TRAF2, ASK1, and MKK7 in the SNpc of the mouse brain was not affected by either MPTP treatment or the deletion of CIB1 gene (Fig. S5).

### Depletion of ASK1 expression by RNAi abolishes the protective effect of CIB1 against MPP^+^-induced neurotoxicity

**Figure 6:**
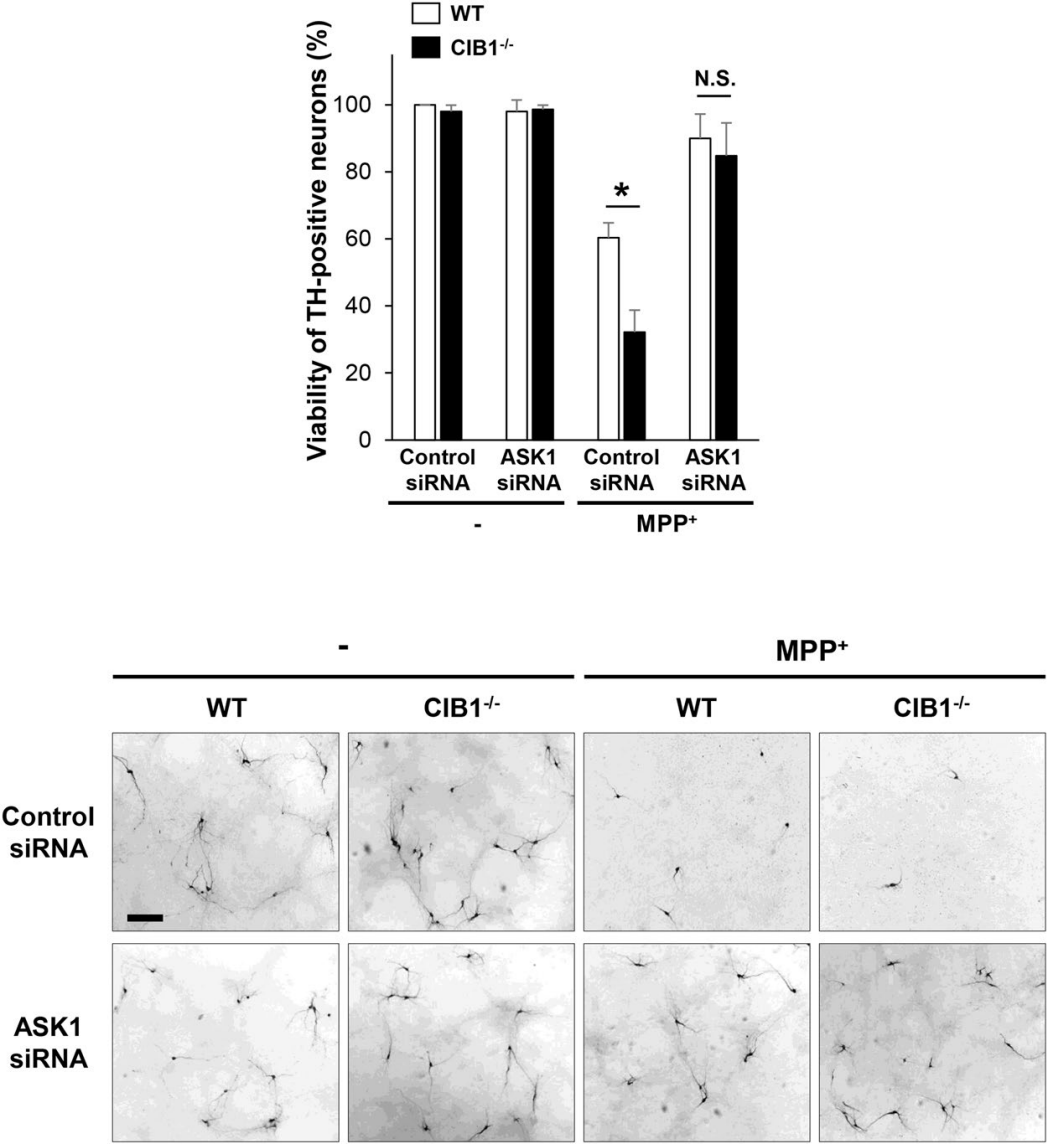
siRNA-mediated depletion of ASK1 abolishes the protective effect of CIB1 on MPP^+^-initiated neurotoxicity. Primary mesencephalic neurons in culture prepared from WT or CIB^−/−^ mice were transfected for 48 h with control (GFP) or ASK1 siRNA. The neurons were left untreated or treated with 200 μM MPP^+^ for 20 h, and then were subjected to immunostaining with anti-TH antibody. The representative images of the stained cells are shown in the bottom panels (scale bar, 100 μm), and the viability of TH-positive neurons is shown in the top panel as a percentage of that of control siRNA-transfected untreated cells. Data are means ± SD of values from three independent experiments. *P < 0.05.

Given that CIB1 directly binds ASK1 and thereby inhibits the MPP^+^-induced activation processes of ASK1, we reasoned that the blockade of ASK1 by CIB1 may be an important part of the mechanism by which CIB1 negatively regulates MPP^+^-mediated toxicity in dopaminergic neurons. If its inhibition of ASK1 is crucial for the neuroprotective function of CIB1, it would be expected that depletion of ASK1 expression in cells might abolish the protective effect of CIB1. We, therefore, examined this possibility with using primary culture of dopaminergic neurons. MPP^+^ treatment reduced cell viability of primary dopaminergic neurons prepared from wild type mouse brain and this neurotoxic effect of MPP^+^ was further aggravated by ablation of CIB1 gene in CIB1^−/−^ dopaminergic neurons (Fig. 6), suggesting that CIB1 negatively regulates MPP^+^-induced death of dopaminergic neurons. Interestingly, the aggravating effect of CIB1 gene ablation on the MPP^+^-induced neurotoxicity was abolished by siRNA-mediated ASK1 depletion in the CIB1^−/−^ neurons.

## Discussion

MPTP is widely used as a neurotoxin in an experimental model of Parkinson’s disease^41^. It produces neuropathological events similar to those observed in Parkinson’s disease patients, including progressive loss of dopaminergic neurons in the SNpc and reduction of striatal dopamine levels^42^. The neurotoxic effect of MPTP depends on the transformation of MPTP to its bioactive form MPP^+^ by monoamine oxidase B in glial cells. MPP^+^ can be taken up into dopaminergic neurons by their dopamine transporters. MPP^+^ inhibits mitochondrial complex I, resulting in decreased production of ATP, increased generation of ROS, and apoptosis^43–45^. The ASK1-JNK1 signaling cascade has been shown to mediate MPTP/MPP^+^-induced neurotoxicity^6,11^. In line with this notion, in the present study, RNAi-mediated ASK1 depletion nullified the MPP^+^-induced death of dopaminergic neurons (Fig. 6), revealing a crucial role of ASK1 in MPTP/MPP^+^ neurotoxicity.

CIB1 is expressed ubiquitously in many cell types and has multiple binding partners, implying its diverse role in a variety of biological events including calcium signaling, proliferation, survival, migration, and adhesion^46^. However, the physiological and pathological functions of CIB1 are not completely understood. Interestingly, public gene expression profiling data indicate that CIB1 is highly expressed in various regions of the brain, including SNpc^47^, implicating its potentially important role in the brain. Noticeably, CIB1 exerts its anti-apoptotic function in neurons by blocking the ASK1-JNK pathway^22^.

In this study, we observed that a genetic deficiency of the CIB1 gene enhances MPTP-induced apoptosis in dopaminergic neurons as well as destruction of the nigral-striatal projections in CIB1^−/−^ mice (Fig. 1), suggesting a protective action of CIB1 against MPTP-induced dopaminergic neurotoxicity. MPTP has been shown to induce microglial activation^48,49^, and chronic microglial activation and inflammation affect the progression of Parkinson’s disease^50^. However, we have ruled out a possibility that the neuroprotective action of CIB1 might result from its modulation of the activation of microglia and astrocytes, because CIB1 was hardly detected in either microglia or astrocytes in the SNpc of mouse brain (Fig. S6A). Additionally, CIB1 does not affect the MPP^+^-induced expression of inflammation-linked cytokines including TNFα and interleukin-6 in microglial BV-2 cells (Fig. S6B). Instead, CIB1 physically associates with ASK1, and thereby prevents ASK1 activation induced by MPP^+^. CIB1 binds to the region containing amino acid residues 378–648 of ASK1 (Fig. 4A). Intriguingly, the CIB1-interacting region of ASK1 is also important for its binding to TRAF2^35,37^. Thus, CIB1 and TRAF2 may compete with each other for an overlapping binding region on ASK1. By binding to ASK1, CIB1 may prevent the recruitment of TRAF2 and subsequent steps of ASK1 activation including the Thr^845^ phosphorylation of ASK1. Moreover, depletion of ASK1 expression by RNAi abolishes the inhibitory action of CIB1 on MPP^+^-induced neurotoxicity (Fig. 6). Together, our results suggest that the inhibition of ASK1 by CIB1 may be the primary mechanism underlying the protective effect of CIB1 against MPTP/MPP^+^-induced neurotoxicity. Interestingly, either the expression levels of CIB1 or its binding to ASK1 in dopaminergic neurons was not changed by MPP^+^ treatment (Figs 3 and 4). These results imply that a portion of ASK1, which forms a complex with CIB1, is unresponsive to MPTP/MPP^+^-induced stress and is kept inactive even under the stress. In this way, CIB1 may prevent the hyper-activation of ASK1 and neurotoxicity during exposure of neurons to MPTP/MPP^+^. Thus, CIB1 may have a buffering effect on MPTP/MPP^+^-induced neurotoxicity. Given a guardian role of CIB1 in MPTP/MPP^+^-induced neurotoxicity, our findings may provide new insights into a potential therapeutic strategy for Parkinson’s disease.

## Materials and Methods

### Cell culture and DNA transfection

Human dopaminergic neuroblastoma SH-SY5Y cells were routinely maintained under a humidified atmosphere of 5% CO_2_ at 37 °C in DMEM (Invitrogen) supplemented with 10% FBS, penicillin (100 U/ml), and streptomycin (100 μg/ml). Transfection of cells was performed with Lipofectamine2000 (Invitrogen) for SH-SY5Y cells or RNAiFect (Qiagen) for primary mesencephalic neuronal cells.

### Primary mesencephalic neuronal cell culture

Primary cultures of ventral mesencephalic dopaminergic neurons were prepared from rat embryos at embryonic day 14 as described previously^2219^. In brief, the neurons were plated at 2.0 × 10^5^ cells per 18 × 18 mm glass coverslip or 4.0 × 10^5^ cells per well in six-well plates pre-coated with poly-D-lysine (50 µg/ml) and laminin (2 µg/ml). The cells were cultured under a humidified atmosphere of 5% CO_2_ at 37 °C in Neurobasal medium (Invitrogen) supplemented with B27 (Invitrogen) and GlutaMax-1 (Invitrogen).

### Plasmids

A full-length human CIB1 cDNA was amplified by PCR from a HeLa Hybrid Hunter Premade cDNA Library (Invitrogen). To construct an expression vector encoding Flag epitope-tagged CIB1 (Flag-CIB1), we amplified CIB1 cDNA by PCR with the primers 5′-ACCAAGCTTATGGGGGGCTCGGGCAGT-3′ (HindIII site underlined) and 5′-ACCGCGGCCGCTCACAGGACAATCTTAAA-3′ (NotI site underlined). The resulting PCR product was digested with HindIII and NotI and inserted into the corresponding sites of the p3xFLAG-CMV-10 vector (Sigma). For bacterial expression of a GST fusion protein of CIB1, CIB1 cDNA was amplified by PCR and cloned into the pGEX4 T vector (Amersham Biosciences). cDNAs for the ASK1 mutants ASK1(1–377), ASK1(378–648), ASK1(649–938), and ASK1(939–1374) were amplified by PCR and cloned into the pET28a vector (Novagen). Mammalian expression vectors encoding HA-ASK1, Myc-ASK1, Myc-JNK1, HA-MKK7, and HA-Trx1were previously described^22,51^.

### RNA interference

The target sequence [sense 5′-AAAGACAGCCTTAGCTTTGAG-3′] for human CIB1 RNAi was selected with the use of the siRNA target finder program of Ambion. The annealed oligonucleotides including sense and anti-sense strands of the target sequence were inserted into the pSUPER.retro vector (OligoEngine). As a control, annealed oligonucleotides containing a target sequence (sense 5′-GGCTACGTCCAGGAGCGCACC-3′) for a GFP shRNA were inserted into the same vector. The nucleotide sequences of the various inserts were confirmed by DNA sequencing. SH-SY5Y cells were transfected with pSUPER.retro vectors encoding either the control (GFP) or CIB1 shRNA, and stable transfectants were selected in the presence of puromycin (0.25 μg/ml). Rat CIB1 siRNA (sense 5′-AAGAGTCACTGCATACCCGAG-3′), Mouse ASK1 siRNA (sense 5′-AATTGCAGTCTGCACAGCCTTTCGG-3′), or control GFP siRNA (sense 5′-GGCTACGTCCAGGAGCGCACC-3′) oligonucleotides were obtained from Invitrogen and introduced into primary rat mesencephalic dopaminergic neurons by transfection for 48 h with the use of RNAiFect (Qiagen).

### Antibodies

Mouse monoclonal antibodies to hemagglutinin (HA) epitope or to Flag epitope were purchased from Roche or Sigma, respectively. Mouse monoclonal antibodies to Myc epitope and rabbit polyclonal antibodies to the Thr^845^-phosphorylated form of human ASK1 were obtained from Cell Signaling Technology. Rabbit polyclonal antibodies to ASK1, TRAF2, and MKK7 were from Santa Cruz Biotechnology. Mouse monoclonal antibodies to JNK1 were obtained from BD Bioscience Pharmingen. Rabbit polyclonal antibodies to tyrosine hydroxylase (TH) were from Chemicon. A polyclonal antibody to CIB1 was generated in rabbits injected with a GST-CIB1 fusion protein.

### Immune complex kinase assays

Immune complex kinase assays were performed as previously described^52,53^. Briefly, cells were lysed in buffer A (20 mM Tris-HCl, pH 7.4, 150 mM NaCl, 1% Triton X-100, 0.5% sodium deoxycholate, 12 mM β-glycerophosphate, 5 mM EGTA, 2 mM sodium orthovanadate, 1 mM phenylmethylsulfonyl fluoride, 2 µg/ml leupeptin, 2 µg/ml aprotinin, 10 mM NaF, 1 mM dithiothreitol), and the soluble fractions (equal amounts of protein) of the lysates obtained by microcentrifugation were subjected to immunoprecipitation with appropriate antibodies. The resulting precipitates were incubated for 30 min at 30 °C in 15 μl of a kinase reaction buffer^53^ in the presence of 1 μCi of [γ-^32^P]ATP and 2 μg of indicated substrate protein. Bacterially expressed GST fusion proteins of MKK6(K82A) and c-Jun(1–79) were used as substrates for ASK1 and JNK, respectively. The reaction mixtures were subjected to SDS-PAGE, and the extent of phosphorylation of the substrate proteins was analyzed with a Fuji BAS 2500 phosphoimager.

### *In vitro* binding assay

GST-CIB1 expressed in *Escherichia coli* was purified with the use of glutathione-conjugated agarose beads (Sigma). ASK1 variants were translated *in vitro* in the presence of [^35^S]methionine with the use of a TNT reticulocyte lysate system (Promega). The ^35^S-labled proteins were incubated at 4 °C for 4 h in a binding buffer^54^ with GST-fused proteins immobilized on glutathione-agarose beads. The bound ^35^S-labeled proteins were eluted from the beads and analyzed by SDS-PAGE and with a Fuji BAS 2500 phosphoimager.

### Immunoblot analysis

Soluble fractions of cell lysates were subjected to SDS-PAGE, and the separated proteins were transferred electronically to PVDF membrane. The membrane was blocked with 5% nonfat milk in washing buffer (50 mM Tris-HCl, pH 8.0, 150 mM NaCl, 0.1% Tween 20), incubated for 1 h at room temperature with primary antibodies in washing buffer containing 1% nonfat milk, washed, and then incubated for 1 h at room temperature with appropriate secondary antibodies conjugated with horseradish peroxidase (Amersham Biosciences). Immune complexes were visualized with the use of enhanced chemiluminescence reagents (Amersham Biosciences).

### Co-immunoprecipitation analysis

Cells were lysed with buffer A, and the lysates were centrifuged at 12,000 × g for 20 min at 4 °C. The resulting supernatants were incubated at 4 °C first for 4 h with appropriate antibodies and then for 1 h in the additional presence of protein G-coupled Sepharose beads (Amersham Biosciences). The resulting precipitates were subjected to SDS-PAGE followed by immunoblot analysis.

### Apoptotic cell death

SH-SY5Y cells expressing either a control or CIB1 shRNA were incubated in the absence or presence of 3 mM MPP^+^ for 20 h. The cells were fixed with 4% formaldehyde and stained with TUNEL using the *In Situ* Cell Death TMR Red kit according to manufacturer’s instructions (Roche). TUNEL-positive cells were analyzed by flow cytometry.

### Cytotoxicity assay for mesencephalic dopaminergic neurons

Primary mesencephalic neuronal cells plated on glass cover slips coated with poly-D-lysine and laminin were incubated in Neurobasal media supplemented with B27 and GlutaMax-1 for 24 h and then transfected for 48 h with either GFP control or rat CIB1 siRNA oligonucleotides. The neurons were incubated in the absence or presence of 200 μM MPP^+^ for 20 h. They were then fixed with 4% paraformaldehyde, permeabilized with 0.2% Triton X-100, and blocked with a solution containing 10% normal horse serum and 0.1% Triton X-100. The cells were incubated at 4 °C overnight with rabbit anti-tyrosine hydroxylase (TH) antibody (1:500; Chemicon), and then for 1 h at room temperature with biotinylated secondary antibodies to rabbit IgG (1:500; Vector Laboratories). Immune complexes were detected with an avidin-biotin immunohistochemical kit (Vector Laboratories). For evaluation of cytotoxicity, the numbers of TH-positive cells were counted in 30 randomly selected fields. Neuronal viability was expressed as a percentage of the viability of untreated cells transfected with the control siRNA. Data are means ± SD of values from three independent experiments.

### MPTP administration for Parkinson’s disease animal model

CIB1 knockout mice were described previously^20^. All animal procedures were approved by the Institutional Animal Care and Use Committee of Korea University, and all methods were carried out in accordance with relevant guidelines and regulations. Routine genotyping was performed on genomic DNA isolated from tail snips of mice with three primers to identify wild-type and null alleles: Primer 1, 5′-AAGACCATCCTGAATCATGC-3′; Primer 2, 5′-AAGTTATGGCGCGCCATCGA-3′; and Primer 3, 5′-TCTTCAGTGACACAGCAAC-3′. Two- to three-month old wild-type (WT) and CIB1^−/−^ mice were used for MPTP treatment. MPTP (Sigma) was administered intraperitoneally using a standard subchronic method. This method consists of injecting MPTP (30 mg/kg) daily for 5 consecutive days and sacrificing at 14 days after the start of injections. Control mice received an equal volume of saline (0.9%).

### Immunohistochemistry

For staining of tyrosine hydroxylase, animals were perfused with PBS and then with 4% paraformaldehyde, and the brains were post-fixed in the same fixative solution for 2 h. Brains were cryoprotected in 30% sucrose and sectioned serially (40 μm). Subsequent immunostaining was performed by the free-floating method. The sections were incubated with anti-tyrosine hydroxylase antibody (1:5000; Chemicon) overnight and then with biotinylated secondary antibodies to rabbit IgG (1:1000; Vector Laboratories) for 1 h at room temperature. Subsequently, immune complexes were detected with an avidin-biotin immunohistochemical kit (Vector Laboratories) and then mounted on gelatin-coated slides, and examined under 40X magnification with a bright-field microscope (Olympus). Tyrosine hydroxylase-labeled neurons were counted with a computerized optical fractionator (Stereo Investigator software v.7; mbf Bioscience, Williston, VT, USA).

## Electronic supplementary material

Supplementary Information
