# Supplementary material for: CIB1 protects against MPTP-induced neurotoxicity through inhibiting ASK1
Source: Sci Rep. 2017 Sep 22;7:12178. doi: 10.1038/s41598-017-12379-3 (PMC5610320; doi:10.1038/s41598-017-12379-3)
Supplement: Supplementary file 1 — Supplementary Information [file 41598_2017_12379_MOESM1_ESM.pdf]

Supplementary informations for

CIB1 protects against MPTP-induced neurotoxicity through inhibiting ASK1

Kyoung Wan Yoon, Hyun-Suk Yang, Young Mok Kim, Yeonsil Kim, Seongman Kang, Woong Sun, Ulhas P. Naik, Leslie V. Parise, and Eui-Ju Choi*

*Corresponding author. E-mail: ejchoi@korea.ac.kr

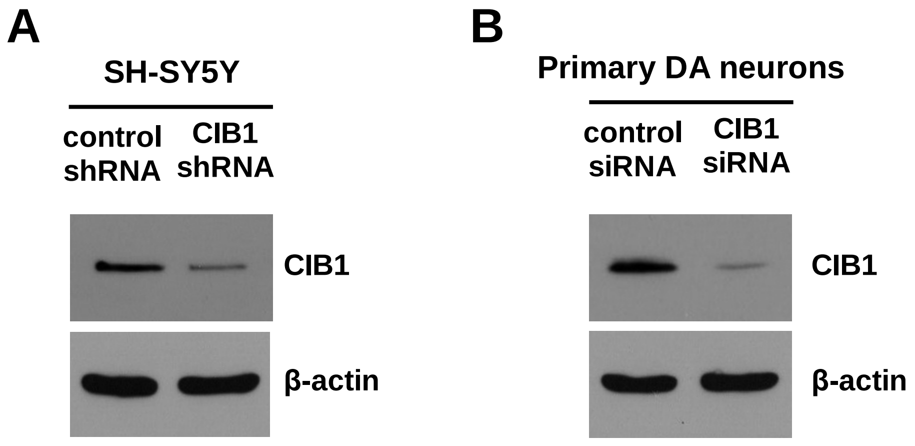

Figure S1. RNAi-mediated depletion of CIB1 in cultured neurons.

SH-SY5Y cells stably expressing either control (GFP) or CIB1 shRNA (A) or primary dopaminergic neurons transfected for 24 h with either control or CIB1 siRNA (B) were lysed. The lysates were subjected to immunoblot analysis with antibodies to CIB1 or to β-actin (a loading control).

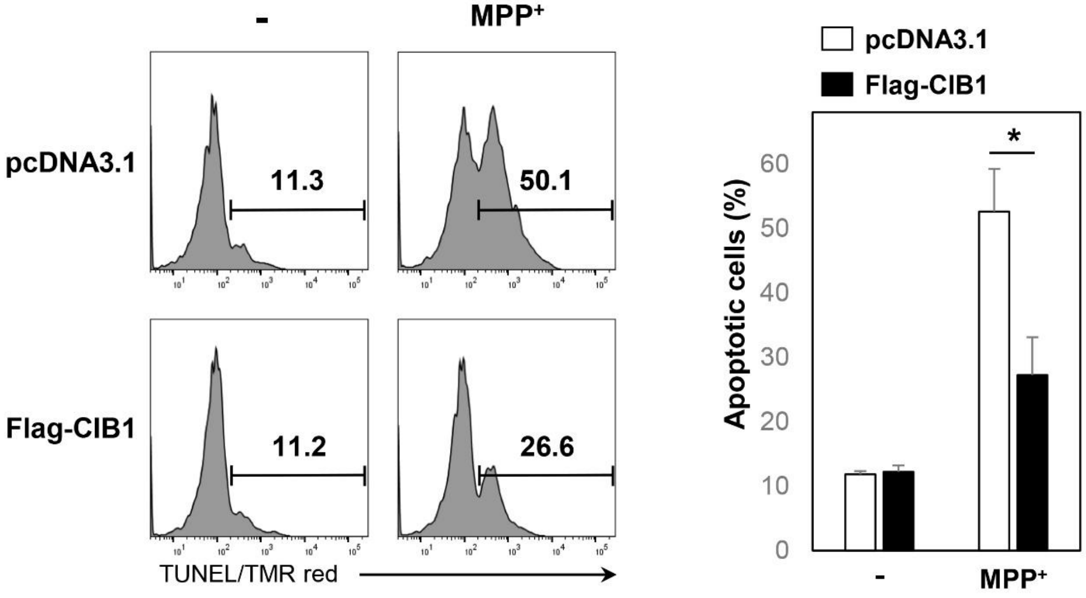

Figure S2. Ectopic CIB1 reduces MPP^+-induced apoptotic cell death in SH-SY5Y cells. SH-SY5Y cells were transfected for 50 h with either an empty vector (pcDNA3.1) or a vector encoding Flag-CIB1, and then incubated for 24 h in the absence or presence of 3 mM MPP^+. The cells were fixed, permeabilized, stained with red fluorescein (TMR red)-labeled TUNEL and analyzed by flow cytometry, as in Fig. 2A. The percentages of TUNEL-positive cells are shown as numbers in the left panel. Representative data of apoptosis are shown in the left panel. The graph in the right panel represents mean ± SD from triplicate experiments (n = 3). $^*P < 0.05$.

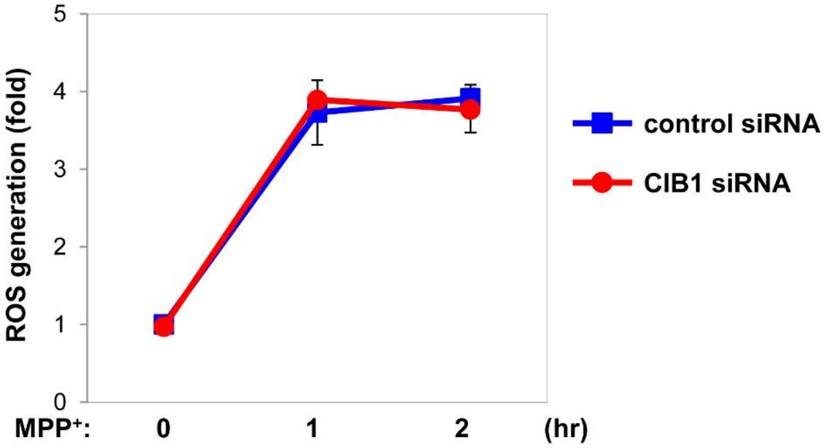

Figure S3. CIB1 does not affect MPP$^+$-induced ROS generation.

SH-SY5Y cells expressing either control or CIB1 shRNA were incubated in the medium containing 3 mM MPP$^+$ for indicated periods of time. Then, the cells were cultivated in fresh serum-free media containing 10 μM dichlorofluorescein diacetate (DCF)-DA for 20 min, and analyzed for ROS production by flow cytometry. ROS production in each sample is shown as the fold of that of control shRNA-expressing untreated cells. Data are means ± SD (n = 3).

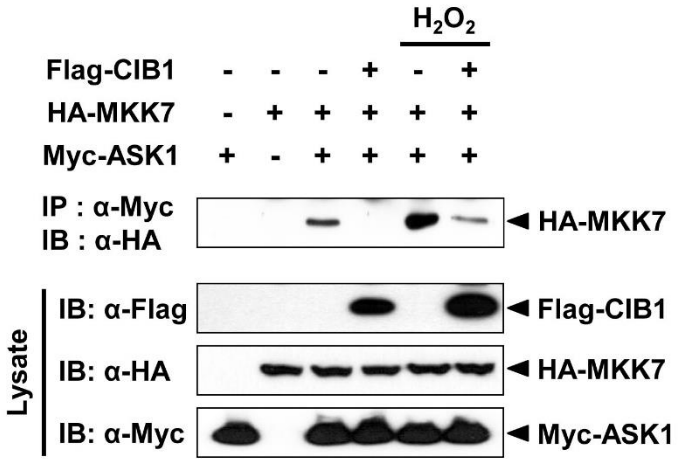

Figure S4. CIB1 block the ROS-induced binding of MKK7 to ASK1.

SH-SY5Ycells expressing either control (GFP) or CIB1 shRNA were incubated in the absence or presence of 1 mM H_2O_2 for 30 min. The cells were lysed, and subjected to immunoprecipitation with anti-Myc antibody. The resulting precipitates were subjected to immunoblot analysis with anti-HA antibody. The cell lysates were also subjected directly to immunoblot analysis with antibodies to Flag, to HA, or to Myc.

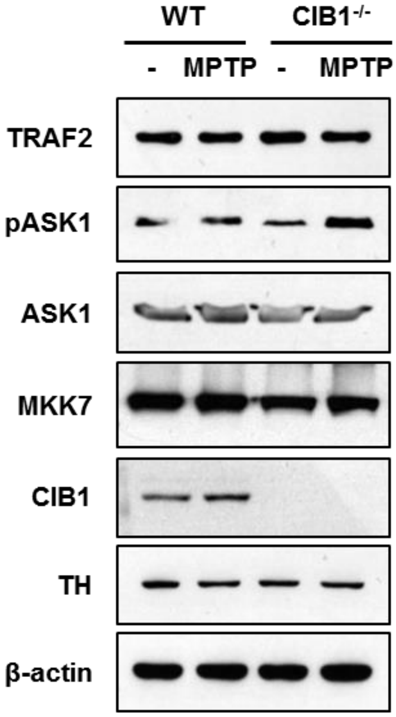

Figure S5. The abundance of TRAF2, pASK1, and MKK7 in the substantia nigra pars compacta of WT and CIB1$^{-/-}$ mice.

WT and CIB1$^{-/-}$ mice were treated with MPTP (30 mg/kg every 24 h, five times) or saline by intraperitoneal injection. Isolated brains were cut using adult mouse brain slicer and the areas of the substantia nigra pars compacta were collected with an 1 mm biopsy punch. The collected tissues were lysed and subjected to immunoblot analysis using indicated antibodies.

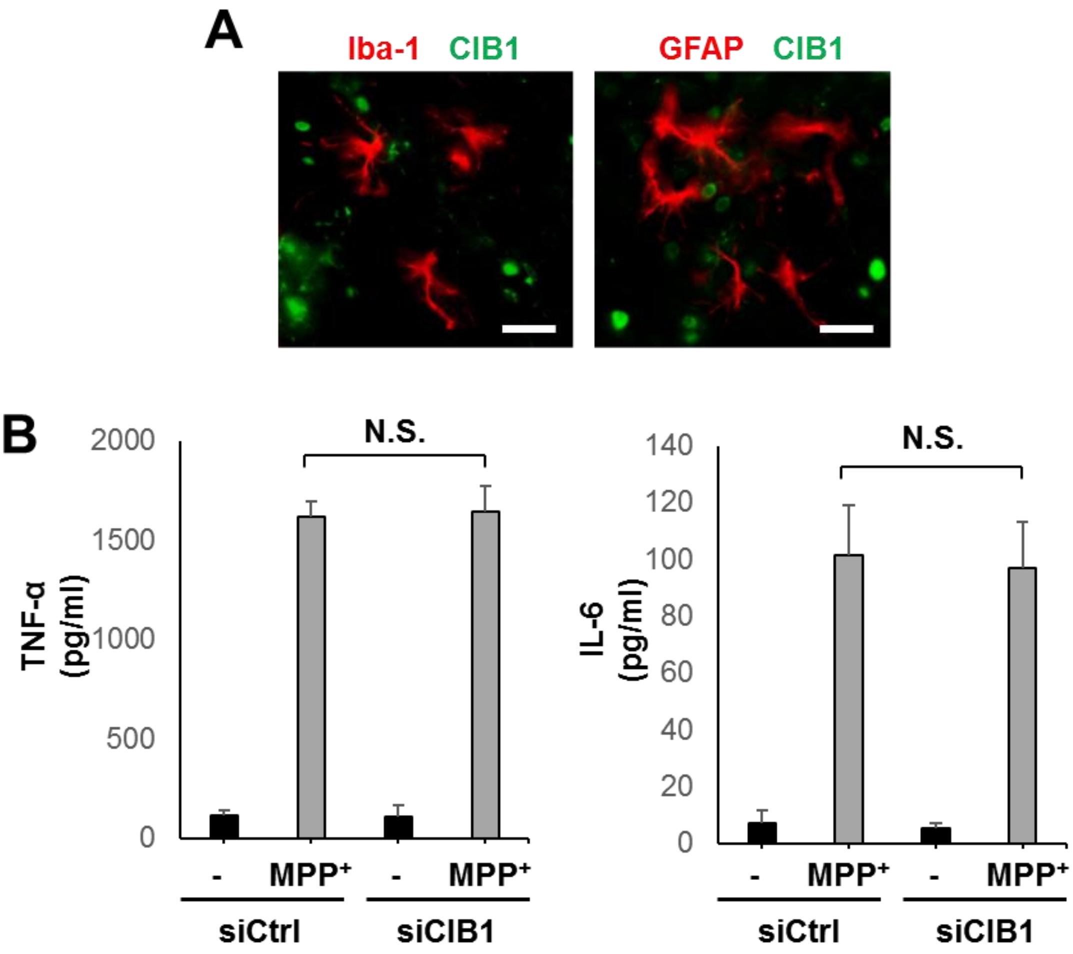

Figure S6. CIB1 does not affect MPP$^+$-induced microglial activation.

(A) Immunohistochemical staining of CIB1 in microglia and astrocytes. Brain of a 3-month-old wild-type mouse was cut into coronal sections of 40 um thickness. The tissue section containing substantia nigra pars compacta was subjected to immunohistochemical staining using anti-Iba-1 or anti-GFAP antibody together with anti-CIB1 antibody. Iba-1- or GFAP-staining (red fluorescence) indicates microglia (left) or astrocytes (right), respectively. CIB1 expression is shown with green signal (scale bar, 25 μm). **(B)** BV2 microglial cells were transfected with control or CIB1 siRNA. After 24 h of transfection, cells were left untreated or treated with 50 μM MPP$^+$ for 24 h. The indicated cytokines in culture media were quantified by ELISA. Data represents mean ± SD (n = 3). N.S., not significant.

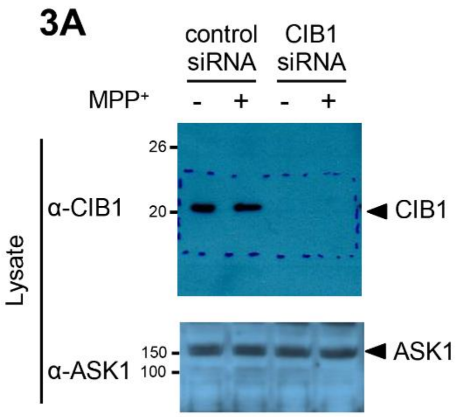

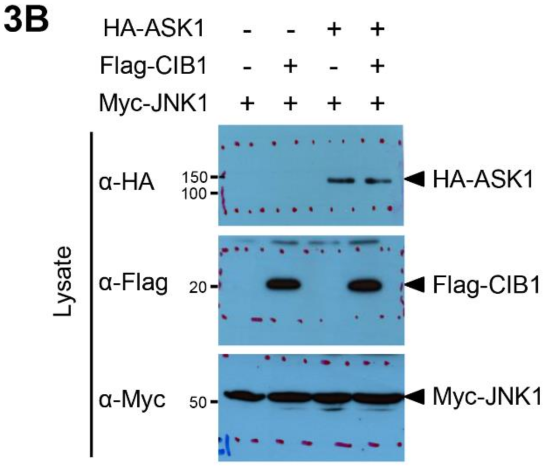

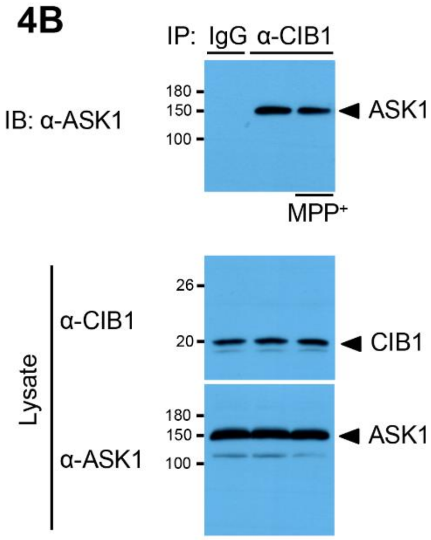

Figure S7. Unprocessed scans of western blots

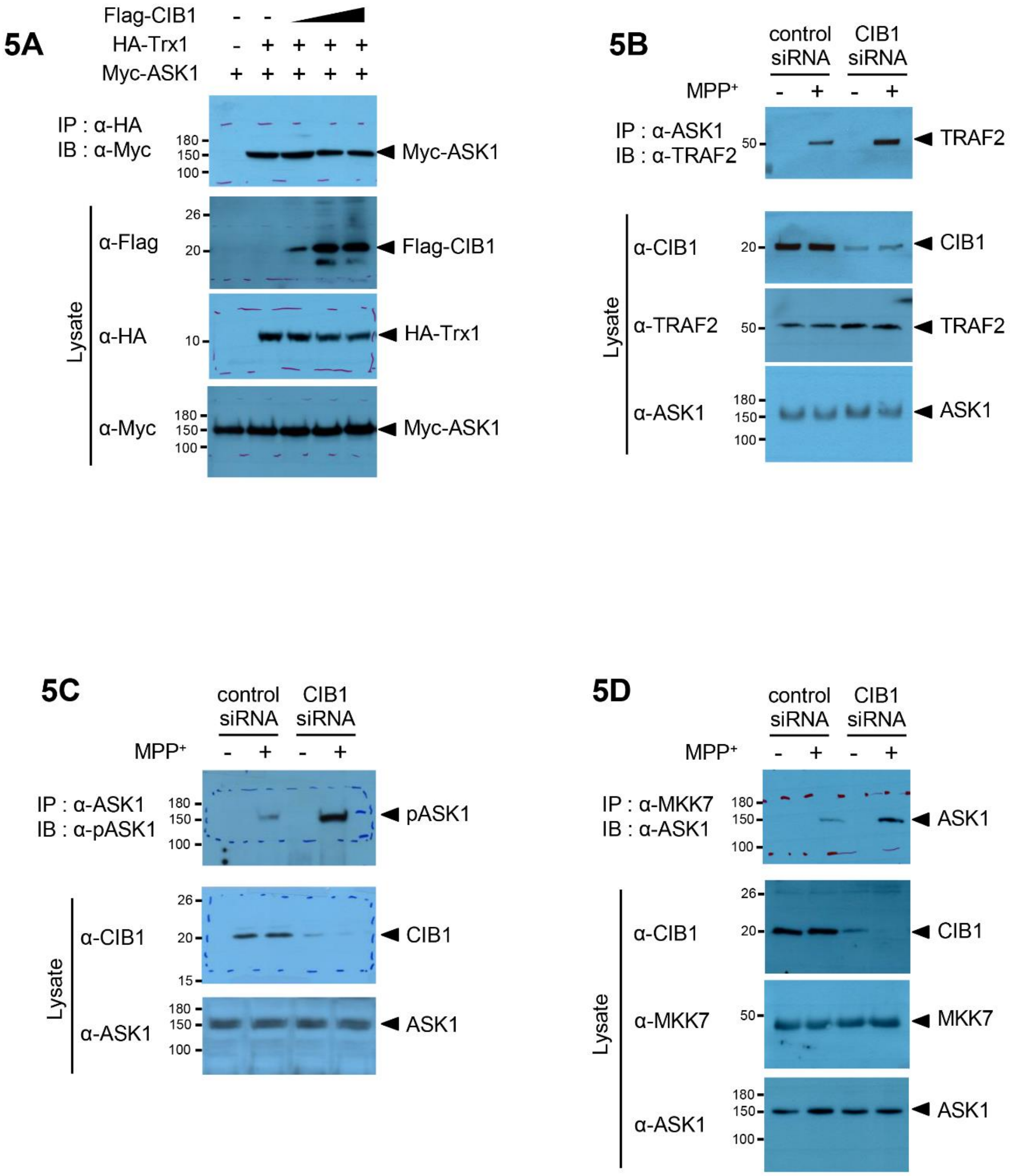

Figure S7. Continued
